# Exosome Nanotechnology in Molecular Medicine: Advances, Applications and Challenges in Gene Therapy

**DOI:** 10.1017/erm.2026.10066

**Published:** 2026-07-13

**Authors:** Elza Karabagh, Babek Alibayov, Adil Allahverdiyev

**Affiliations:** 1 V.Y. Akhundov Scientific Research Medical Preventive Institute, Baku, Azerbaijan; 2 School of Advanced Technologies and Innovation Engineering, Western Caspian University, Baku, Azerbaijan; 3 Department of Biotechnology, Baku Engineering University, Baku, Azerbaijan

**Keywords:** drug delivery, exosomes, gene therapy, molecular medicine, nanotechnology

## Abstract

**Background:**

Gene therapy has emerged as a transformative approach for treating diseases that are caused by genetic defects, including cancer, inherited metabolic disorders and immunodeficiencies. However, its clinical success depends critically on the availability of safe, efficient and non-immunogenic delivery systems. Conventional viral vectors, while highly effective, are associated with immunogenicity, oncogenic risk and high production costs. Non-viral alternatives offer improved safety but they have lower transfection efficiency and limited stability. Exosomes are naturally secreted, endosome-derived nanovesicles ranging from 30–150 nm in diameter and have emerged as promising next-generation carriers for gene and drug delivery. Their inherent biocompatibility, low immunogenicity, ability to cross biological barriers and capacity to transport diverse molecular cargo including nucleic acids, proteins and small molecules make them particularly attractive as therapeutic platforms.

**Methods:**

This review summarizes the biogenesis, molecular composition and classification of exosomes and examines their applications in siRNA/miRNA delivery, CRISPR/Cas9 gene editing, mRNA therapy, cancer treatment and vaccine development. Special attention is given to engineered, plant-derived and stem cell-derived exosomes, as well as current cargo-loading strategies and commercial therapeutic platforms.

**Results:**

Despite their promise, significant challenges remain, including low cargo-loading efficiency, batch heterogeneity, limited scalability and the absence of standardized manufacturing and regulatory frameworks.

**Conclusions:**

Future research must address these barriers to accelerate the clinical translation of exosome-based therapeutics.

## Graphical abstract

**Figure:**
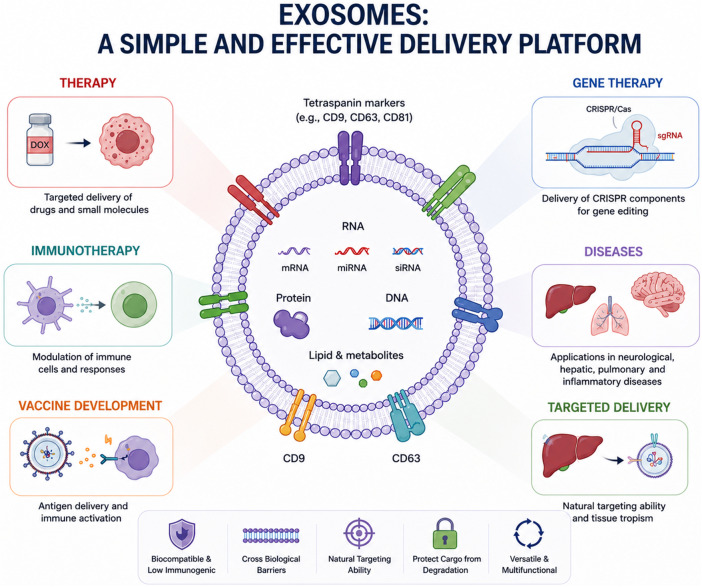

## Introduction

Gene therapy involves the transfer of genetic material into cells for the purpose of modifying cellular functions, and this process can be achieved through various methods and vectors. The genetic material delivered to cells may take the form of DNA, RNA or proteins (Refs 1–3). The primary goal of gene therapy is to enable the treatment of diseases by correcting genetic defects, introducing new genes or modulating gene expression (Ref. 2). In particular, gene therapy is regarded as a promising approach for conditions that are difficult to manage or cannot be fully cured by conventional methods, including cancer, inherited metabolic disorders and immunodeficiencies (Refs 2, 3).

**Figure 1.**
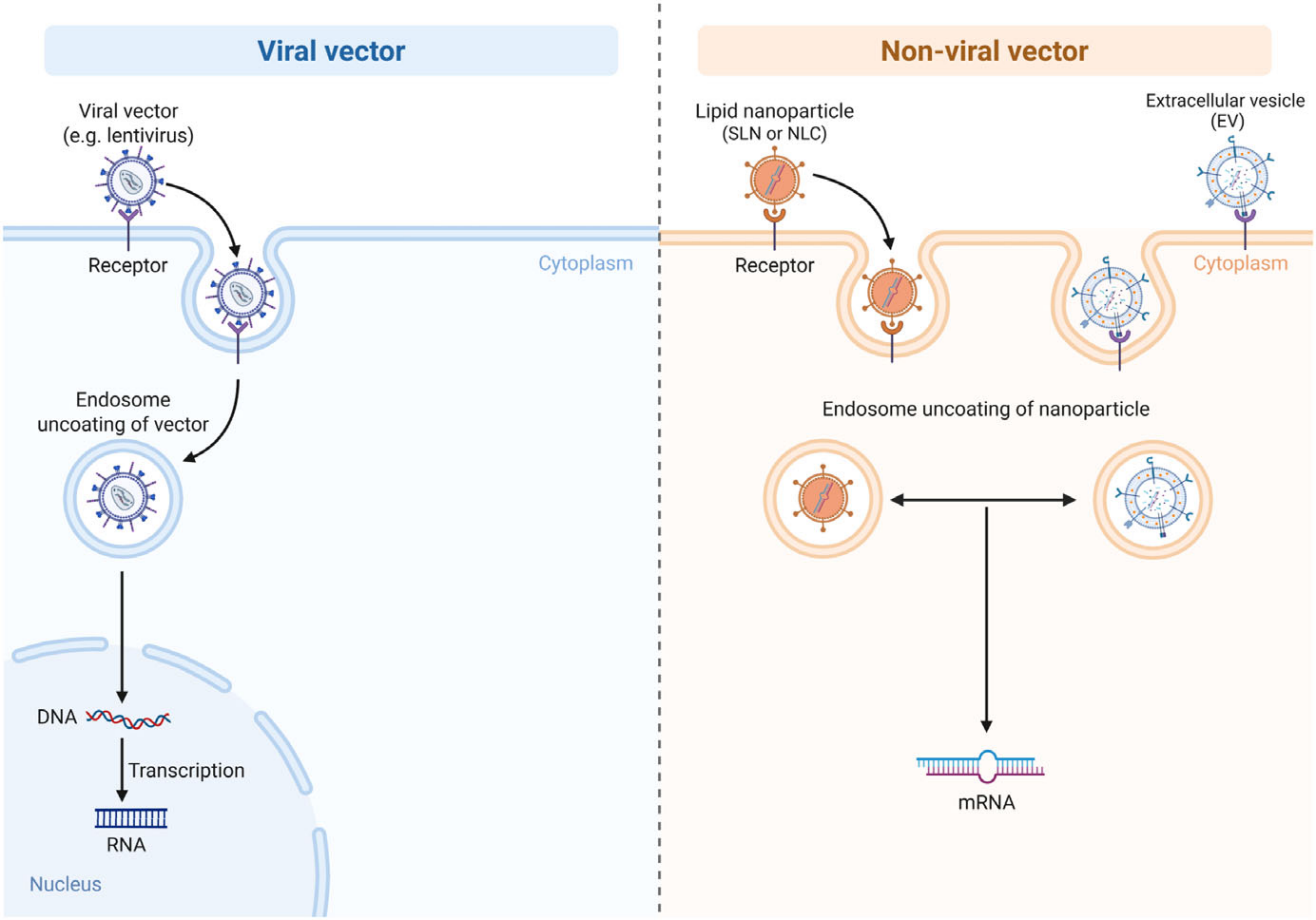
Viral and non-viral vectors used for gene delivery. Schematic comparison of viral vectors (e.g., lentivirus) and non-viral nanocarriers such as lipid nanoparticles, solid lipid nanoparticles (SLN), nanostructured lipid carriers (NLC) and extracellular vesicles (EVs), including exosome-enriched vesicle systems. Both systems undergo receptor-mediated uptake and endosomal uncoating, leading to intracellular release of genetic material (DNA or mRNA). Figure 1. long description.

Delivering genetic material into cells is a critical step for the success of gene therapy. However, the cell membrane represents a significant barrier for negatively charged nucleic acids. Moreover, because genetic materials are highly sensitive within the human body, their protection is essential for effective therapy. For this purpose, messenger RNAs (mRNAs) and plasmid DNAs are widely used, which can be delivered through both viral and non-viral vectors (Refs 2–8) (Figure 1). Viral vectors including adeno-associated viruses (AAVs), retroviruses, lentiviruses (LVs), adenoviruses (Ads) and herpes simplex viruses, which provide high transfection efficiency and long-term gene expression. Nevertheless, they carry notable drawbacks, such as high cost, risk of immune response and potential oncogenic effects (Ref. 8). Non-viral vectors, such as cationic polymers, cationic lipids, engineered polymers, nanoparticles and naked DNA, are generally safer and less immunogenic and can accommodate larger genetic payloads. However, their transfection efficiency and the duration of gene expression are typically lower compared to viral vectors (Refs 2, 9).

Significant progress has been made toward developing gene-transfer systems that are more efficient, less toxic and less immunogenic, while also enabling long-term transgene expression. One of the key challenges for the successful clinical application of gene therapy is the ability to tightly and consistently regulate gene expression as needed (Ref. 10). The major obstacle to clinical translation remains the lack of an effective, non-immunogenic delivery system. The efficacy of gene therapy primarily depends on the efficiency of nucleic acid delivery to the target site and the precision of the vectors used (Ref. 11).

Extracellular vesicles (EVs), and particularly exosomes, are currently among the most widely utilized nanocarriers in the field of drug delivery. EVs are lipid bilayer-delimited particles naturally released by cells that have generated profound therapeutic interest. Crucially, EVs comprise distinct subpopulations classified by their biogenesis pathways, including microvesicles, apoptotic bodies and exosomes. This review focuses specifically on exosomes, operationally defined as nano-sized, endosome-derived vesicles typically ranging from 30–150 nm in diameter. While the broader scientific literature occasionally uses the terms EVs and exosome interchangeably, the present paper maintains a strict focus on exosomes as a distinct physiological entity, evaluating their unique capacities as gene and drug delivery vectors.

## Gene therapy

Over the years, gene therapy has advanced substantially, with numerous clinical trials conducted worldwide. According to the updated global database reported by Ginn et al. (Ref. 7), approximately 3,900 gene therapy clinical trials had been completed, approved or remained ongoing worldwide as of December 2023, a significant increase compared to earlier reports: by January 2005, 1,020 trials had been recorded; by 2007, the number had reached 1,340; and by 2012, it had risen to 1,800 (Table 1).

**Table 1.** Gene therapy products applied in medical practice Table 1. long description.

Product	Regulatory agency	Approval year	Diseases	Mechanism of action	Limitations/Challenges
Luxturna (voretigene neparvovec)	FDA	2017	Inherited retinal dystrophies	Delivery of functional RPE65 gene via AAV2	High cost; applicable only to ocular diseases
Zolgensma (onasemnogene abeparvovec)	FDA	2019	Spinal muscular atrophy (SMA)	Replacement of SMN1 gene via AAV9	Risk of immune response; extremely high cost
Strimvelis	EMA	2016	ADA-SCID	Ex vivo gene transfer to patient’s hematopoietic stem cells using a retroviral vector	Complex ex vivo procedure; limited availability
Kymriah (tisagenlecleucel)	FDA	2017	B-cell acute lymphoblastic leukemia; diffuse large B-cell lymphoma	CAR-T therapy: modification of autologous T-cells targeting CD19	Cytokine release syndrome; complex manufacturing process
Yescarta (axicabtagene ciloleucel)	FDA	2017	Large B-cell lymphoma	CAR-T therapy: modification of autologous T-cells targeting CD19	Toxicity; high cost
Gendicine	CFDA (China)	2003	Head and neck squamous cell carcinoma	Delivery of p53 gene via adenoviral vector	Limited efficacy in solid tumors
Imlygic (talimogene laherparepvec)	FDA	2015	Melanoma	Oncolytic HSV-1 expressing GM-CSF	Only for local injection; restricted to accessible lesions

Currently, CRISPR-Cas9 gene-editing technology enables precise, efficient and relatively cost-effective modifications in the human genome. In recent years, CRISPR-Cas9 has attracted significant attention in the fields of science and engineering, as it provides unprecedented accuracy, efficiency and flexibility for altering, deleting or replacing specific genes (Refs 12–14). The development of this technology has led to remarkable advances in treating a wide range of diseases. However, the vectors currently used for CRISPR-Cas9 delivery remain limited by issues of immunogenicity. Compared with earlier methods such as zinc finger nucleases (ZFNs) and transcription activator-like effector nucleases (TALENs), CRISPR-Cas9 has greatly simplified genetic modifications and has shown promising results in the treatment of various genetic disorders, including neurodegenerative diseases and cancers.

Gene therapy can be broadly categorized into two main types: somatic gene therapy and germline gene therapy. Somatic gene therapy targets the genes of specific cells within the body such as blood cells to treat or alleviate diseases. The modifications made through this approach affect only the treated individual and are not passed on to future generations. In contrast, germline gene therapy targets reproductive cells, such as eggs and sperm, with the aim of preventing the transmission of genetic disorders to subsequent generations.

### Advantages and disadvantages of gene therapy vectors

Viral vectors exhibit high efficiency in delivering genetic material to host cells, resulting in robust gene expression. They can integrate into the host genome, allowing for long-term expression of therapeutic genes, and they protect genetic material from biological degradation, enhancing stability. However, viral vectors can activate immune responses, which may limit their effectiveness and cause adverse effects. Their integration into the host genome carries the risk of insertional mutagenesis, potentially leading to unwanted genetic alterations. Additionally, viral vectors have limitations regarding the size and structural flexibility of the genetic material they can carry.

Non-viral vectors, in contrast, are generally less likely to trigger immune responses, making them safer for repeated administration. They offer high structural flexibility and can be easily tailored for specific applications. The production of non-viral vectors is usually simpler and more cost-effective than that of viral vectors. Nevertheless, non-viral vectors are typically less efficient at delivering genes into cells, which may limit their therapeutic effectiveness. They are also more susceptible to degradation in biological environments, reducing stability and overall efficacy, and they often require complex delivery strategies to overcome physiological barriers and achieve effective gene transfection.

## Gene therapy vectors

Gene therapy can be divided into two main approaches: *in vivo* and *ex vivo*. in vivo gene therapy involves the direct delivery of therapeutic genes into the patient’s body, targeting specific tissues or cells. *Ex vivo* gene therapy entails the modification of cells outside the patient’s body, followed by reinfusion of the genetically modified cells back into the patient. Each method has its own distinct processes, advantages and challenges.

The delivery of genes into cells is a fundamental step in gene therapy, involving the transfer of therapeutic genes to target cells. To enhance the efficiency and safety of gene delivery, various methods have been developed, including viral and non-viral approaches such as bacterial vectors, hybrid vectors and lipid-based systems. Viral vector-mediated delivery is currently the most widely used approach. Viral vectors have become central to gene therapy owing to their high transduction efficiency and ability to target diverse tissues. The most commonly used viral vectors include LVs, Ads and AAVs, which together account for over 80% of approved viral-based gene therapy products (Ref. 15). To date, a total of 14 *ex vivo* and 21 *in vivo* vector-based gene therapy products have received market approval, of which 29 are viral vector-based and 6 are non-viral vector-based therapies (Table 1) (Ref. 15).

**Figure 2.**
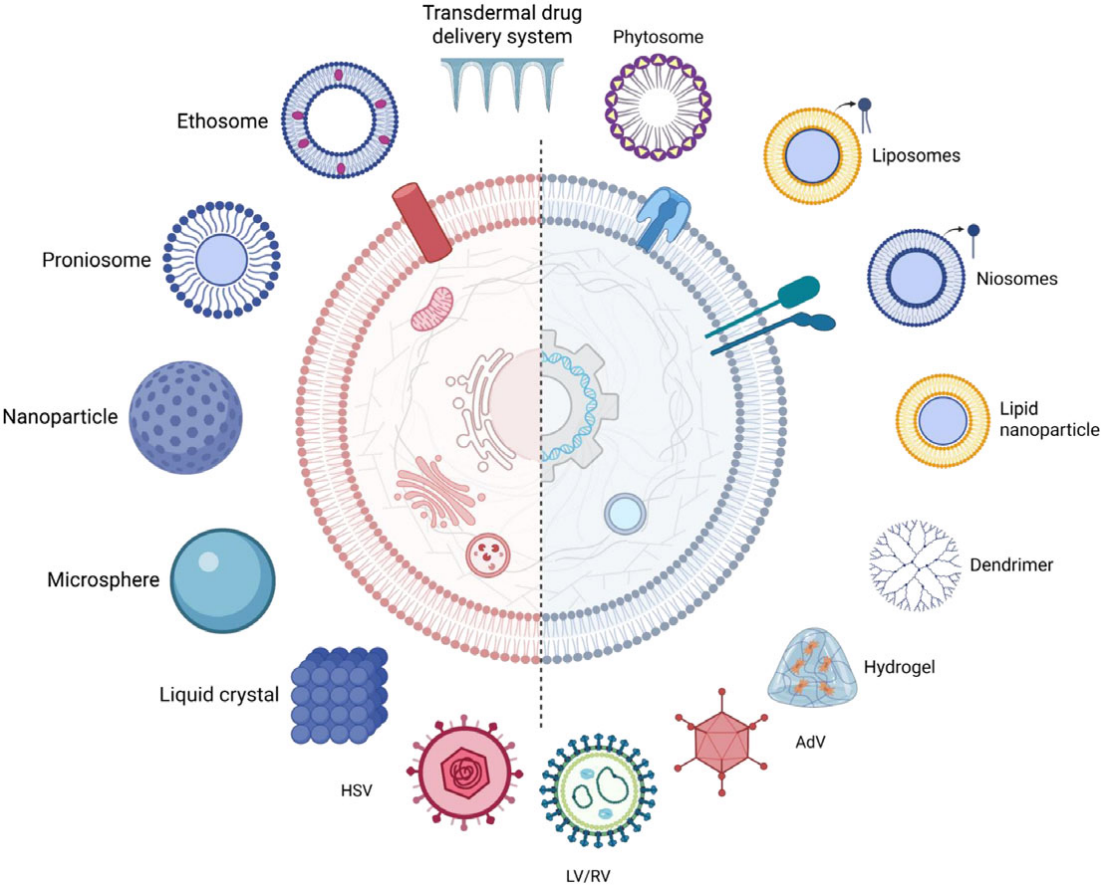
Overview of nano- and micro-scale delivery platforms. A broad range of delivery systems-including ethosomes, proniosomes, nanoparticles, microspheres, liquid crystals, liposomes, niosomes, lipid nanoparticles, dendrimers, hydrogels and viral vectors (HSV, LV/RV, AdV)-depicted around a central cell interface. These platforms are utilized for targeted and controlled drug or gene delivery. Figure 2. long description.

The strong *in vivo* infectivity of viruses makes them highly effective therapeutic tools (Figure 2). An ideal viral vector for gene therapy should meet several criteria: (a) the capacity to carry therapeutic genes of a sufficient size – herpesviruses, for instance, can package large DNA inserts; (b) ease of packaging and production – AAVs are notable for their production efficiency and stability; (c) replication-deficiency and safety – AAV vectors are suitable for clinical use due to their favourable safety profile and low immunogenicity and (d) high in vivo transduction efficiency – LVs and Ads exhibit particularly high transduction efficiency, which is critical for effective gene therapy (Refs 16, 17).

### Lentiviral vectors

The lentiviral genome consists of several key structural and regulatory elements essential for replication and pathogenicity. These viruses are enveloped, single-stranded RNA viruses with a diameter of approximately 100 nm (Ref. 17). Lentiviral vectors (LVs) are widely recognized in gene therapy, particularly for *ex vivo* applications, due to their high efficiency. LVs can transduce both dividing and non-dividing cells, which is crucial for targeting a broad range of cell types, and they integrate into the host genome, enabling long-term therapeutic gene expression (Refs 17, 18, 19). These vectors are especially effective in the modification of T cells, leading to immune responses against cancer via chimeric antigen receptors (CARs) or cloned T-cell receptors. CAR-T cell therapies developed using LVs have demonstrated significant clinical success, particularly in the treatment of B-cell malignancies, and represent the first regulatory-approved genetically engineered cell therapy using LVs (Ref. 20). Approved LV-based *ex vivo* gene therapies include three hematopoietic stem cell transplantation (HSCT) therapies and eight CAR-T therapies for refractory hematologic malignancies and other diseases (Ref. 16).

### Adenoviral vectors

Adeno-associated virus (AAV) possesses a single-stranded DNA genome of approximately 4.7 kb, encapsulated within a non-enveloped, icosahedral capsid with an outer diameter of about 20 nm (Ref. 21). In the early 1990s, Ad vectors were first used for in vivo gene therapy, delivering and expressing the *α*-1 antitrypsin (*A1AT*) gene in mouse hepatocytes and lung tissues, an important milestone that demonstrated the potential of Ad vectors for treating human monogenic diseases (Ref. 22). However, Ad vectors are known to trigger strong immune responses in humans. Upon administration, Ad vectors rapidly activate the innate immune system, which can lead to cytokine storms, disseminated intravascular coagulation, thrombocytopenia, and hepatotoxicity, thereby increasing the risk of morbidity and mortality (Ref. 23).

In clinical studies, various viral vectors are employed for in vivo gene delivery. Retroviruses are used for achieving long-term gene expression, while vectors such as HSV and VSV are effective in the treatment of neurologic disorders and cancers. Vectors derived from MVA, arenaviruses, Sendai virus and measles virus are particularly promising for vaccine development and oncolytic therapy due to their strong immunogenic potential (Refs 16, 17).

### Bacterial vectors

Specific bacterial strains, such as *Listeria monocytogenes* or *Salmonella enterica*, can be engineered to carry therapeutic genes. Once these bacteria enter host cells, they release the genetic material, enabling expression of the desired protein within eukaryotic cells. Bactofection has potential applications in cancer therapy, where bacteria can deliver genes that induce apoptosis in tumour cells or enhance immune responses, and in vaccine development, where bacteria can deliver antigens directly to immune cells (Refs 24, 25). An alternative strategy involves the use of prokaryotic expression systems to produce therapeutic proteins without modifying the host genome, with protein expression regulated by external signals. This approach is particularly useful for producing proteins such as enzymes, hormones or antibodies for therapeutic purposes. Bacteria can also be applied in biosensor development and vaccine production, where proteins can be delivered without altering the host genome (Refs 25, 26).

Although viral vectors are widely employed for delivering therapeutic gene fragments, they have certain limitations, including cancer risk, immunogenicity, broad tropism and restricted gene packaging capacity. In contrast, non-viral vectors produced through chemical synthesis or physical methods can overcome many of these limitations (Ref. 16).

### Lipid nanoparticles

Lipid nanoparticles (LNPs) have emerged as a transformative non-viral vector platform for gene therapy, enabling the delivery of nucleic acids such as DNA and RNA. LNPs exhibit low immunogenicity and cytotoxicity, protect fragile genetic material from degradation and facilitate delivery to target cells, ensuring that the genetic cargo reaches its intended site of action. They are used to deliver therapeutic genes to correct genetic defects and offer potential treatment strategies for a variety of genetic disorders (Ref. 27). Typically, LNPs consist of four main components: a cationic or ionizable lipid, a helper lipid, a polyethylene glycol (PEG)-lipid and cholesterol. Each component influences the LNPs’ delivery efficiency, fluidity, colloidal stability and structural integrity (Ref. 16).

Another targeted delivery approach exploits the specific binding of N-acetylgalactosamine (GalNAc) to the asialoglycoprotein receptor (ASGPR) on hepatocytes, enabling receptor-mediated endocytosis for targeted delivery and cellular uptake (Ref. 28). The number and arrangement of GalNAc residues are critical: tri- or tetravalent multivalent GalNAc structures enhance binding affinity and uptake efficiency, thereby increasing the therapeutic effectiveness of the conjugates. Several GalNAc-siRNA drugs have been approved by the FDA, demonstrating the clinical potential of this delivery system for silencing specific genes in the treatment of various liver diseases (Ref. 29).

Polyplex gene therapy involves the use of polyplexes-complexes formed via electrostatic interactions between nucleic acids (e.g., DNA or siRNA) and cationic polymers (Ref. 17). These non-viral vectors deliver genetic material to target cells and offer a safer alternative to viral vectors. Polyplexes typically enter cells through endocytosis, and strategies such as the “proton sponge effect” facilitate endosomal escape into the cytoplasm. Incorporating targeting ligands into polyplexes can enhance specificity toward certain cell types, thereby improving therapeutic outcomes. Polyplex gene therapy holds significant potential for the treatment of various diseases, although ongoing research is necessary to further enhance the efficiency and safety of these systems (Ref. 30).

To provide a more systematic and evidence-based comparison, major gene-delivery platforms including exosomes, AAVs, lentiviral vectors and LNPs are evaluated using a common set of translational and functional metrics (Table 2). These metrics include payload capacity, biodistribution and clearance, immune response, functional delivery efficiency, repeat dosing potential and manufacturing scalability. Importantly, each platform’s advantages are presented alongside their inherent limitations and boundary conditions, reflecting the context-dependent nature of their therapeutic performance rather than implying universal superiority of any single system.

**Table 2.** Comparative characteristics of major gene delivery platforms Table 2. long description.

Parameter	AAV	Lentivirus (LV)	Adenovirus (AdV)	LNP	Polyplex	Exosome
**Payload capacity**	~4.7 kb (ssDNA)	~8 kb	~36 kb	Flexible (siRNA, mRNA, DNA)	Flexible (siRNA, mRNA)	Limited; low loading efficiency (1%–10%)
**Transduction/delivery efficiency**	High	Very high	High (transient)	High (liver-tropic in vivo)	Moderate	Low – moderate (preclinical)
**Immunogenicity risk**	Low – moderate; pre-existing Ab possible	Moderate (insertional risk)	High (cytokine storm risk)	Low (innate activation possible)	Moderate (cytotoxicity at high dose)	Low (autologous); moderate (allogeneic)
**Biodistribution/clearance**	Serotype-dependent; liver, CNS tropism	Broad; integrates into host genome	Broad; rapid clearance	Primarily liver; PEGylation extends t½	Liver/spleen accumulation; rapid renal clearance	Liver/spleen sequestration; MPS clearance
**Repeat dosing**	Limited (neutralizing Ab)	Limited (immune sensitization)	Not suitable	Feasible	Feasible (cytotoxicity dose-dependent)	Feasible (autologous); low immunogenicity
**Scalability/GMP manufacturing**	Moderate; established GMP platforms	Complex; patient-specific (ex vivo)	Moderate; established platforms	High; robust GMP pipelines (e.g., COVID mRNA vaccines)	Moderate; chemical synthesis	Low; no standardized GMP framework
**Insertional mutagenesis risk**	Very low	High	None (episomal)	None	None	None
**Clinical approval status**	Multiple approved products (e.g., Luxturna, Zolgensma)	Multiple approved (CAR-T, HSCT)	Limited (primarily vaccine use)	Multiple approved (Onpattro, Comirnaty)	Approved (GalNAc-siRNA: Inclisiran, Givosiran)	None approved; Phase I–II trials ongoing

*Note*: AAV: adeno-associated virus; AdV: adenovirus; LV: lentiviral vector; LNP: lipid nanoparticle; MPS: mononuclear phagocyte system; GMP: good manufacturing practice; Ab: antibody; t½: half-life. Data represent general consensus from published literature; individual values may vary by serotype, formulation, or clinical context.

## Exosomes and their biogenesis

Exosomes are a subtype of EVs, initially described as a mechanism for cells to dispose of waste products (Ref. 31). Although exosomes are classified as a subtype of EVs, the terminology used in the literature remains heterogeneous due to limitations in current isolation and characterization techniques. According to the Minimal Information for Studies of Extracellular Vesicles (MISEV) guidelines proposed by the International Society for Extracellular Vesicles (ISEV), the term “small extracellular vesicles” (sEVs) is often preferred when vesicles are classified primarily by size rather than definitive endosomal origin (Ref. 32). However, because many published studies rely on enrichment methods such as ultracentrifugation, precipitation-based isolation, filtration or size-exclusion chromatography, complete separation of exosomes from other small EV populations is often not achievable. Consequently, the term “exosomes” continues to be widely used operationally in the literature to describe vesicle preparations enriched in endosome-derived small vesicles. In this review, the term “exosomes” is retained because the primary focus is on their biogenesis, biological properties and therapeutic applications, while acknowledging that some cited studies may involve heterogeneous small EV populations (Refs 32, 33).

**Figure 3.**
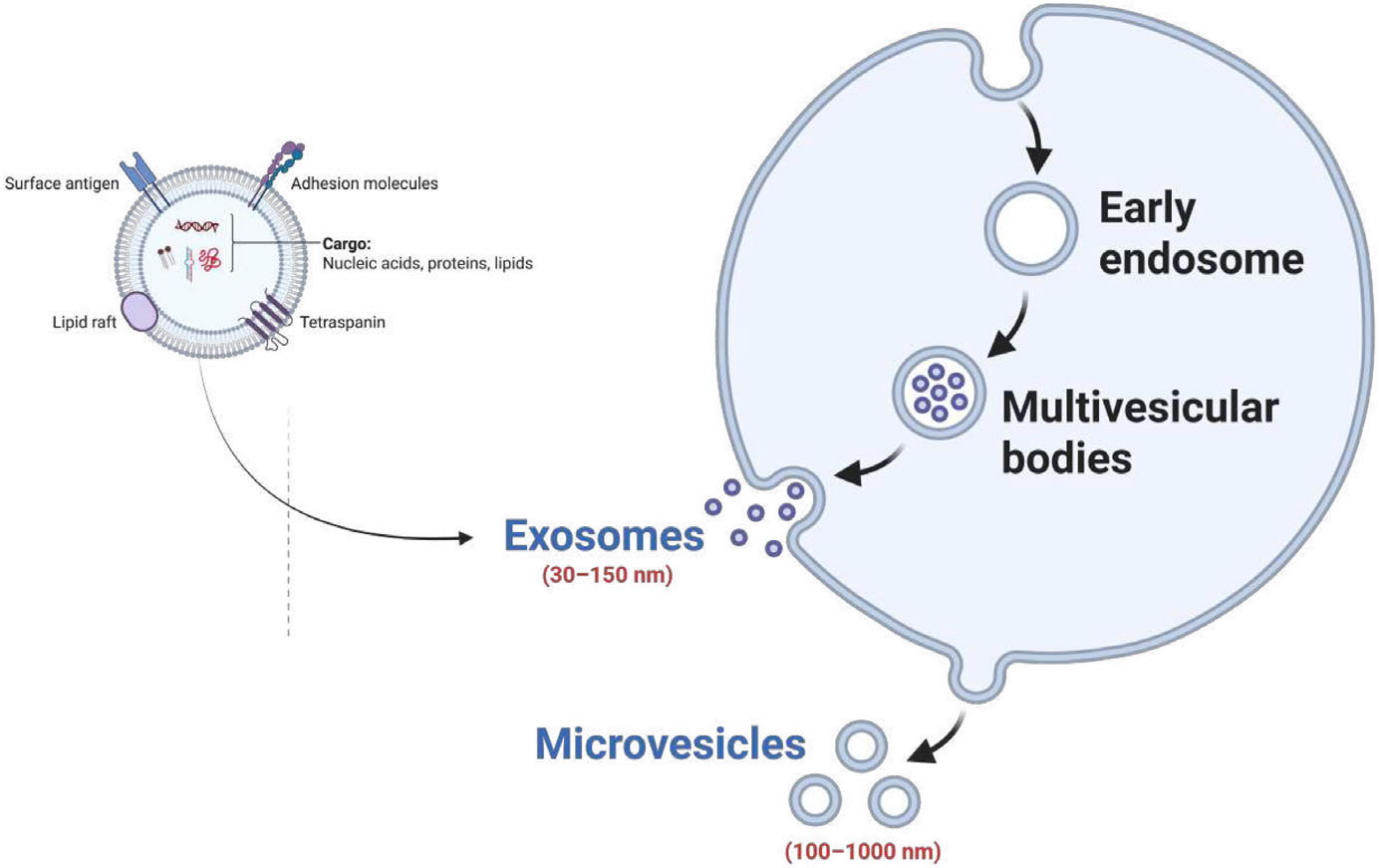
Biogenesis and characteristics of exosomes within the extracellular vesicle (EV) family. Illustration of the formation of extracellular vesicles. Exosomes (30–150 nm) originate from multivesicular bodies within early endosomes, while microvesicles (100–1000 nm) bud directly from the plasma membrane. Figure 3. long description.

Recent studies have demonstrated that exosomes play a crucial role in intercellular signalling and the exchange of biological information. They transport nucleic acids, functional proteins, lipids and various metabolites to target cells, acting as key mediators of cellular communication. Exosome formation and biogenesis are associated with the endosomal pathway and occur in three main stages: (1) early endosome formation, in which invagination of the plasma membrane via endocytosis generates early endosomes; (2) maturation to late endosomes, in which early endosomes mature into late endosomes that invaginate inward to form multivesicular bodies (MVBs) containing numerous intraluminal vesicles (ILVs) (Ref. 34) (Figure 3) and (3) exosome release, in which MVBs either fuse with lysosomes for degradation or merge with the plasma membrane, releasing their internal vesicles into the extracellular environment as exosomes. This final stage is a key step in intracellular material recycling and exosomal secretion (Ref. 34).

During exosome biogenesis and secretion, the Endosomal Sorting Complex Required for Transport (ESCRT) complex plays a crucial role, regulating endosomal membrane budding, protein sorting and vesicle formation. The activity of various ESCRT sub-complexes has differential effects on exosome production: reduction of certain sub-complexes impairs exosome formation, while activation of others enhances secretion, indicating that exosome biogenesis is a highly regulated, multi-level and dynamic process. Exosomes are secreted by virtually all cell types, and the intensity of secretion varies depending on the cell type – cancer-derived cells exhibit relatively stable but lower levels of secretion, whereas normal cells release exosomes more intensively.

Structurally, exosomes are single-membrane, nano-sized vesicles with diameters ranging from approximately 30–150 nm, sharing the same topology as the plasma membrane (Ref. 35). They are characterized as organelle-like structures enriched with specific proteins, lipids, nucleic acids and glycoconjugates. The density of exosomes typically ranges from 1.13–1.19 g/mL in a sucrose density gradient, which is one of the distinguishing features that sets them apart from other EVs (Ref. 35).

### Molecular composition

Exosomes are enclosed by a lipid bilayer, and their composition varies depending on their cellular origin. Their bioactivity is determined not only by their protein and nucleic acid content but also by their lipid composition. The lipid profile of exosomes is rich in phosphatidylserine (PS), phosphatidic acid, cholesterol, sphingomyelin (SM), arachidonic acid, prostaglandins and leukotrienes, which contribute to structural stability and mechanical resilience. The main molecular components of exosomes can be grouped as follows:

- Membrane proteins: Tetraspanins (CD9, CD63, CD81), Rab proteins and integrins are characteristic exosomal markers that play critical roles in exosome recognition, binding to target cells and intracellular transport (Ref. 36).
- Lipid components: Cholesterol, sphingomyelin and various phospholipids maintain the membrane stability and biological functionality of exosomes (Ref. 36).
- Genetic material: Exosomes carry mRNA, microRNA (miRNA), long non-coding RNA (lncRNA) and sometimes DNA fragments. These molecules are involved in intercellular gene regulation, signalling and phenotypic reprogramming of target cells. Recent studies also indicate that exosomal circular RNAs (circRNAs) play significant roles in gene expression regulation and oncogenic processes.
- Cytoplasmic components: Enzymes and signalling proteins within exosomes can modulate various biochemical and signalling pathways in recipient cells (Ref. 36).

Thus, exosomes are not only structurally complex but also serve as key participants in various biological processes, including intercellular communication, regulation of gene expression and the pathogenesis of diseases.

### Classification of exosomes

**Figure 4.**
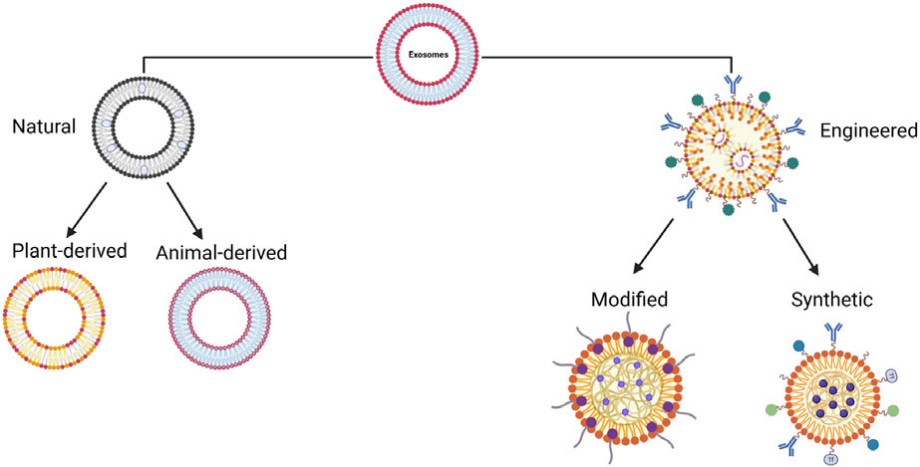
Classification of natural and engineered exosomes. Exosomes are categorized into natural (plant-derived and animal-derived) and engineered types. Engineered exosomes include modified vesicles and fully synthetic exosome-mimetic nanocarriers designed to enhance targeting, loading capacity and therapeutic function.

Exosomes can generally be classified into three main categories: natural exosomes, modified exosomes and synthetic exosomes (Figure 4). Natural exosomes are further divided into animal-derived and plant-derived subgroups, with animal-derived exosomes distinguished as tumour-derived or normal cell-derived. Many cell types, including epithelial cells, endothelial cells, mesenchymal stem cells (MSCs), macrophages, dendritic cells and various immune cells, naturally secrete exosomes under physiological conditions. The biological functions of these exosomes vary significantly depending on their cellular origin. Macrophage-derived exosomes, for example, can influence the tumour microenvironment and exhibit antitumor properties, whereas MSC-derived exosomes are promising drug carriers and therapeutic agents. The origin of exosomes determines their composition, biological functions and drug-loading potential, which explains why exosomes from different sources may have distinct therapeutic mechanisms.

Exosomes can be isolated from various biological fluids such as blood, serum, milk and urine. Due to their biological origin and natural carrier properties, exosomes are considered promising nanosystems for drug delivery and have been successfully used, for example, to deliver paclitaxel. One of the primary functions of exosomes is to act as messengers in intercellular communication, influencing target cells through their molecular cargo and regulating both physiological and pathological processes. Exosomes secreted by neural precursor cells transfer key miRNAs (e.g., miR-21a) to target cells, promoting neurogenesis while facilitating signalling between epithelial and mesenchymal cells to contribute to tissue development (Ref. 37). Conversely, exosomes released from cancer and adipose cells modulate disease processes by activating inflammatory and fibrotic signalling pathways, respectively.

## Exosome-based drug and gene delivery systems

### Plant- and stem cell-derived exosomes

The therapeutic potential of exosomes largely depends on their ability to interact with target cells and effectively deliver genetic material. Exosome–target cell interactions occur through two primary mechanisms: (1) cellular uptake via endocytosis or macropinocytosis, allowing exosomes to deliver their cargo directly into the cell; and (2) ligand–receptor interactions on the exosome membrane, which can activate signalling cascades in the recipient cell. Once inside the cell, exosomes fuse with endosomal membranes, releasing their internal cargo into the cytoplasm of the target cell, enabling functional utilization of the genetic material (Refs 31, 34, 35).

Through their bioactive components, exosomes can influence recipient cells in multiple ways: by directly stimulating target cells via ligands on the exosomal surface; by transferring activated receptors to the recipient cell, thereby modulating signalling pathways and by delivering functional proteins, lipids and RNAs, enabling epigenetic and functional reprogramming of the target cells (Refs 35, 36). The biological and therapeutic properties of exosomes – including low immunogenicity, intrinsic stability, and the ability to cross biological barriers – make them ideal nanosystems for the delivery of functional cargos. Compared to synthetic liposomes, biologically derived exosomes enable more efficient transport of bioactive molecules and demonstrate higher efficacy in cellular uptake and delivery of therapeutic agents (Refs 37, 38).

Interest in exosomes as drug carriers stems from their unique properties. Their biocompatible and biodegradable membrane structure minimizes immune responses and toxicity risks. Due to their small size, exosomes can overcome biological barriers, remain in circulation for extended periods and enhance drug delivery efficiency (Refs 35, 37, 39). Moreover, exosomes possess the ability to recognize and target specific cells and tissues, which improves therapeutic efficacy and reduces side effects. Recent studies demonstrate that exosomes can serve as versatile and multifunctional nanosystems for drug delivery, carrying proteins, nucleic acids and small-molecule drugs (Figure 3) (Ref. 39).

#### A) Delivery of Small Molecules

Several studies have shown that exosomes can be effective carriers for therapeutic small molecules, including curcumin, paclitaxel and doxorubicin (DOX). When curcumin is loaded into exosomes, for instance, it achieves higher plasma concentrations compared to free curcumin and reduces inflammation, improving survival in septic shock mouse models (Ref. 39).

#### B) Delivery of Proteins

Exosomes have been explored for transporting large biomolecules, including enzymes, antigens and cytoskeletal and membrane proteins. An exosome platform carrying the antioxidant enzyme catalase, for example, was delivered intranasally to brain cells in a Parkinson’s disease mouse model and successfully taken up by neurons (Ref. 39).

#### C) Delivery of Genetic Material

Exosomes protect nucleic acids from degradation, making them ideal carriers in gene therapy. Delivery of siRNA and miRNA via exosomes is generally more efficient and less toxic than conventional transfection methods. CRISPR/Cas9 gene-editing technology has shown great potential for precise and flexible gene modification; however, existing delivery systems are often limited by immunogenicity. Naturally derived exosomes have shown promise for delivering CRISPR/Cas9 components to target cells. Additionally, exosomes can be bioengineered to improve the loading efficiency of large molecules such as Cas9 proteins, enhancing target gene inhibition and the expression of delivered genetic material in recipient cells (Ref. 39).

### Plant-derived exosomes

Plant-derived exosomes (PDEs) are plant extracellular nanovesicles that share several structural and functional similarities with mammalian EVs but differ in biogenesis pathways, lipid composition, protein markers and cargo signatures. Unlike mammalian exosomes, plant-derived vesicles lack canonical mammalian exosomal markers such as CD9, CD63 and CD81, and their mechanisms of vesicle biogenesis and cellular uptake remain less well characterized. Exosomes isolated from various plants – including carrot, ginger, lemon, cabbage, wild berries, orange, tomato, grapefruit and tea leaves – exhibit diverse therapeutic properties, including anti-inflammatory, antioxidant and anticancer activities. PDELVs also show significant potential for targeted drug delivery and other therapeutic applications. Compared to animal-derived exosomes, PDELVs offer several advantages: a wider variety of raw materials, ease of mass production and lower extraction costs. Since they can be obtained from commonly consumed plants, their production is both efficient and abundant, making them promising endogenous carriers for drug delivery. Like artificial liposomes, lipid nanoparticles, and other natural EVs, PDEs function as lipid-based carriers, delivering drugs to target cells and generating therapeutic effects *in vivo* (Ref. 40).

### Stem cell-derived exosomes

Exosomes derived from human mesenchymal stem cells (hMSCs), induced pluripotent stem cells (hiPSCs) and human embryonic stem cells (hESCs) differ in terms of their biogenesis mechanisms, molecular cargo and effects on target cells (Ref. 41). Among various cell sources, hMSC-derived exosomes are readily accessible and possess well-defined therapeutic effects. Studies have shown that exosomes derived from bone marrow-derived mesenchymal stem cells exhibit good tolerance to repeated administrations in the treatment of graft-versus-host disease (GVHD) and do not induce significant adverse effects. GVHD is a serious complication occurring during stem cell or bone marrow transplantation when donor immune cells recognize the recipient’s tissues as foreign and attack them; donor T cells can damage the recipient’s organs, particularly the skin, liver and gastrointestinal system. Exosomes could represent a promising and safe novel tool for the treatment of GVHD and other inflammation-related diseases that are difficult to manage.

In addition, exosomes derived from milk and bacteria are also utilized. Human breast milk is a natural nutritional fluid containing milk fat globules, immune cells and soluble proteins such as immunoglobulins, cytokines and antimicrobial peptides. Recent studies indicate that breast milk is rich in exosomes, which are important for regulating infant immunity, preventing necrotizing enterocolitis (NEC) and shaping the gut microbiota. Bacterial-derived vesicles (BEVs) are nanosized membrane vesicles secreted by Gram-positive and Gram-negative bacteria, and they are considered potential carriers for stimulating the immune system, delivering antigens and transporting bioactive molecules into cells. They are widely employed in vaccine development as well as in microbiome and infection research.

## Exosome-based gene delivery systems

Surface modifications of exosomes can enhance their tropism to target cells, biodistribution and therapeutic efficacy. Exosomes are small lipid membrane-enclosed vesicles that can deliver their cargo, such as drugs, proteins or genetic material, to target cells either by fusion with the target cell membrane or via endocytosis. Their internal microdomain structures provide a protective environment for transported biomolecules, thereby increasing their bioactivity. Exosomes can be tailored for specific therapeutic applications through various modifications. For instance, dendritic cell-derived exosomes modified with pH-sensitive GALA peptides become activated in acidic environments, enabling targeted drug delivery to specific cells. Exosomes enriched with biotin and LA proteins further enhance targeted delivery and drug transport potential. This strategy shows particular promise for antigen delivery to tumour cells, inflammation reduction and treatment of neurodegenerative diseases.

Despite their promising biological properties, exosomes should not be considered universally superior to conventional viral or non-viral delivery systems. Compared with AAVs and lentiviral vectors, exosomes generally demonstrate lower immunogenicity and improved biocompatibility, particularly for repeated administration. However, viral vectors still provide higher transduction efficiency and more consistent long-term gene expression in many clinical settings. Similarly, LNPs currently offer superior scalability, manufacturing reproducibility and nucleic acid loading efficiency, which contributed substantially to the rapid clinical deployment of mRNA therapeutics (Ref. 42). In contrast, exosome-based systems face ongoing challenges related to heterogeneity, purification, cargo-loading efficiency, quality control and large-scale production (Ref. 43). Therefore, the therapeutic advantages of exosomes are highly context-dependent and may vary according to cargo type, target tissue, administration route, donor-cell origin and manufacturing methodology.

Exosome-mediated CRISPR/Cas delivery has demonstrated promising preclinical efficacy by improving intracellular protection of ribonucleoprotein complexes while reducing the immunogenicity associated with viral vectors. However, efficient loading of Cas9 proteins, guide RNAs and donor templates remains technically challenging. Exosome-mediated mRNA delivery has likewise emerged as a biologically compatible alternative to LNPs, offering enhanced nuclease protection, reduced innate immune activation and potentially improved tissue tropism. Nevertheless, manufacturing scalability, batch reproducibility and loading consistency remain major barriers to clinical translation. By protecting mRNA within the intracellular environment, exosomes increase translation efficiency and direct therapeutic molecules to target cells without eliciting immune responses providing substantial opportunities for the development of personalized mRNA therapies.

### Cargo-loading strategies

Exosome-based cargo-loading strategies differ significantly depending on the physicochemical nature of the therapeutic payload, and methodological rigor requires classification based on cargo type, including small RNAs (siRNA/miRNA), messenger RNA (mRNA), plasmid DNA and proteins or ribonucleoprotein (RNP) complexes. Each cargo class presents distinct constraints in terms of loading efficiency, vesicle membrane stability and intracellular functional delivery.

For small RNA molecules such as siRNA and miRNA, loading is commonly achieved via electroporation, passive incubation or transfection of donor cells. However, reported loading efficiency is often overestimated due to RNA aggregation or precipitation induced by electrical pulses, which can be misinterpreted as successful encapsulation. A critical distinction must therefore be made between surface-associated RNA and true intraluminal encapsulation protected from enzymatic degradation.

mRNA loading into exosomes is technically more challenging due to molecular size and susceptibility to degradation. Methods such as donor-cell engineering or lipid-based transfection of parental cells are generally more reliable than direct loading approaches. Nevertheless, efficient cytosolic release remains a limiting step, as endosomal entrapment significantly reduces translational efficiency.

Plasmid DNA loading is typically inefficient in exosome systems because of size constraints and nuclear delivery requirements. In most cases, observed DNA association reflects external binding or co-isolated nucleic acid contamination rather than functional encapsulation, requiring rigorous nuclease protection and transcriptional activity assays for validation.

Protein and RNP loading strategies, including Cas9-sgRNA complexes, rely on protein engineering, affinity-based loading motifs or parental cell expression systems. While functional delivery has been demonstrated in selected models, protein stability and retention within vesicles remain major limiting factors.

Across all cargo classes, true functional delivery should be validated using orthogonal assays that confirm intracellular activity rather than mere uptake or fluorescence signal detection.

## Exosomes: Vaccine development and commercial therapeutic applications

Exosomes possess a range of therapeutic applications in biomedicine, and their translation into clinical products is increasingly being shaped by advances in GMP manufacturing, scalable purification strategies and regulatory-compliant quality control systems. Researchers are focusing on utilizing exosomes for the development of cell-free vaccines against cancer and other infectious diseases (Refs 44, 45, 46), driven by their high biocompatibility, low toxicity and minimal immunogenicity. Exosomes can deliver antigens to recipient cells; however, their *in vivo* distribution is influenced by non-specific uptake and clearance by the mononuclear phagocyte system, which may limit effective targeting efficiency. Exosomes derived from mesenchymal stem cells exhibit pro-angiogenic, anti-inflammatory and immunomodulatory properties and provide a novel approach for antigen delivery, facilitating the rapid production of multivalent protein-based vaccines. Such vaccines aim to combat emerging SARS-CoV-2 variants and limit viral spread. Clinical trials of the STX vaccine have demonstrated promising results regarding effective protein delivery. Exosome-based therapeutic drug delivery platforms are currently in preclinical and clinical research stages, with several companies worldwide having developed exosome-based products (Table 3).

**Table 3.** Key companies and their products Table 3. long description.

Company	Platform/Source	Product/Technology	Therapeutic area	Cargo type	Administration route	Development stage	CMC/QC information
Aruna bio	Neural stem cells	AB126 exosome platform	Neurological disorders	Native EV cargo	IV (preclinical models)	Preclinical	Proprietary isolation; limited QC disclosure
Ciloa	Recombinant exosomes	Engineered exosome vaccine platform	Viral diseases	Antigen/protein	Injection	Preclinical/early clinical	Engineered loading; scalable recombinant system
Evox therapeutics	Engineered exosomes	DeliverEX™ platform	Rare genetic disorders	RNA/protein therapeutics	IV	Preclinical/clinical pipeline	GMP-oriented scalable EV engineering platform
Carmine therapeutics	RBC-derived EVs	REGENT® platform	Gene therapy	RNA/DNA cargo	IV	Preclinical	Enucleated RBC source reduces oncogenic risk
Direct biologics	MSC-derived EVs	ExoFlo™	Inflammatory diseases	Native MSC secretome	IV	Clinical use (limited indications)	Batch variability noted; MSC-based GMP production
Aethlon medical	Filtration-based system	Hemopurifier®	Cancer, viral infections	EV depletion/immune modulation	Extracorporeal	Clinical device stage	Device-based CMC framework
Kimera labs	MSC-derived EVs	XoGlo®	Wound healing, skin repair	Growth factors + EVs	Topical/injection	Commercial/clinical use	MSC-conditioned media; limited standardization
Capricor therapeutics	Cardiac MSC EVs	Exosome platform	Duchenne muscular dystrophy	Protein/RNA cargo	IV	Clinical trials	GMP MSC expansion + EV isolation pipeline
Codiak biosciences	Engineered EVs	engEx™ platform	Cancer, CNS diseases	Engineered RNA/protein	IV	Clinical trials	Defined engineering + QC biomarker panels
Exopharm Pty Ltd	Purified EVs	LEAP technology	Multiple indications	Purified EV fractions	IV/topical	Preclinical/early clinical	Scalable purification + EV enrichment QC
ReNeuron	Stem cell EVs	ExoPrO platform	Neurodegenerative diseases	Native EV cargo	IV	Clinical pipeline	Stem cell-derived GMP manufacturing

Good Manufacturing Practice (GMP), Chemistry, Manufacturing and Controls (CMC), and translational standardization represent critical bottlenecks in exosome-based therapeutics. Source selection is a primary determinant of product quality, with commonly used cell sources including mesenchymal stem cells, dendritic cells, immune cells and engineered producer cell lines, each contributing distinct cargo profiles and safety implications. Scalable purification remains challenging due to overlapping size distributions with other EVs and protein aggregates, necessitating multi-step isolation workflows combining ultrafiltration, size exclusion chromatography and density gradient centrifugation.

Key critical quality attributes (CQAs) from a CMC perspective include particle size distribution, surface marker expression (e.g., CD9, CD63, CD81), protein and RNA cargo composition, sterility, endotoxin levels and functional potency assays. Release criteria for clinical-grade exosomes typically require stringent validation of identity, purity, potency and safety, although no universally standardized regulatory framework currently exists. Dose definition also remains a major translational limitation, as exosome therapeutics are commonly quantified using particle number, total protein content or lipid concentration, none of which have direct equivalence to pharmacologically active dose. Safety concerns include donor-cell-derived oncogenic or immunomodulatory cargo, batch-to-batch variability, and unintended biodistribution to reticuloendothelial organs such as the liver and spleen. Despite increasing commercial activity, the transition of exosome therapeutics into late-stage clinical development therefore requires improved GMP standardization, validated potency assays and harmonized regulatory guidelines.

## Challenges in gene and drug delivery via exosomes

Although exosomes present significant potential as gene and drug delivery systems, their application is limited by several technical and biological challenges. Biological limitations primarily arise from intrinsic heterogeneity, donor-cell-dependent molecular composition, rapid clearance by the mononuclear phagocyte system and non-specific biodistribution. Exosomes frequently accumulate in the liver, spleen and macrophage-rich tissues, limiting effective delivery to target organs. Additionally, batch-to-batch variation in lipid, protein and nucleic acid composition may influence biological activity, reproducibility and safety.

Engineering limitations are largely associated with therapeutic cargo-loading efficiency, preservation of vesicle integrity during modification, scalable manufacturing, purification reproducibility and quality control. A primary challenge is the low efficiency of therapeutic cargo loading: existing methods such as electroporation, sonication and chemical-based approaches often result in low loading percentages and may damage the exosomal membrane. This is particularly problematic when incorporating large molecules such as plasmid DNA or long mRNA (Ref. 47). Another critical limitation concerns cargo stability within exosomes: loaded genetic material and drug molecules are susceptible to physical and chemical factors during preparation, storage and transfer. Freezing exosomes, for instance, can increase membrane permeability and potentially lead to leakage of encapsulated cargo (Ref. 48). When exosomes enter target cells via endocytosis, a portion of the cargo may be degraded in lysosomes, meaning that without effective endosomal escape mechanisms, the bioactivity of the cargo can be compromised (Ref. 49).

The isolation and purification methodologies commonly used, such as differential ultracentrifugation and filtration, lack the high-resolution specificity required to completely isolate endosome-derived exosomes from other co-purified elements, including small plasma membrane-derived microvesicles, retrovirus-like particles and non-vesicular protein aggregates. This technical limitation results in heterogeneous, mixed exosome or sEV fractions, compromising batch-to-batch uniformity (Ref. 50). Standardized characterization approaches, including evaluation of canonical exosomal markers, such as CD9, CD63, CD81, TSG101, and Alix, are therefore essential for improving reproducibility and translational reliability (Refs 32, 33).

A major methodological limitation in exosome cargo studies is the frequent conflation of cargo association with true encapsulation and functional delivery. Many loading studies rely solely on fluorescence labelling or PCR-based detection, which cannot distinguish between surface-bound, co-precipitated, or intraluminal cargo. This is particularly evident in electroporation-based siRNA loading, where vesicle aggregation and RNA precipitation can lead to false-positive loading efficiency measurements. Rigorous validation therefore requires the use of nuclease or protease protection assays, detergent-disruption controls, density-gradient purification and functional readouts such as gene knockdown, protein translation or enzymatic activity in recipient cells.

Regarding targeting specificity, exosomes can be naturally home to certain cells, but non-specific uptake is widespread: exosomes may be sequestered by the liver, spleen and macrophages during circulation, reducing the amount of therapeutic cargo delivered to intended tissues. Surface functionalization with specific ligands, antibodies or peptides can enhance targeting efficiency, but this adds additional preparation steps and complicates standardization (Ref. 51–56). Concerning immune responses, exosomes are generally biocompatible; however, allogeneic exosomes may contain foreign antigens that can activate the host immune system, induce proinflammatory cytokine release and reduce carrier lifespan. The heterogeneity of exosomes presents further limitations, as their specific lipid, protein and nucleic acid composition depends on the donor cell type, and exosomes derived from cancer cells may carry oncogenic factors, necessitating additional safety considerations.

An additional limitation in evaluating exosome biodistribution and targeting efficiency arises from methodological constraints in tracking approaches. Common labelling strategies, including fluorescent dyes, lipophilic tracers, and nanoparticle-based imaging probes may suffer from signal dissociation, dye aggregation or transfer to non-vesicular components, leading to potential overestimation of true exosome localization. Radiolabelling and bioluminescence approaches, while more sensitive, may alter vesicle properties or fail to distinguish intact exosomes from degraded components. These technical limitations highlight the need for standardized, multimodal tracking approaches and careful interpretation of reported targeting efficiency *in vivo*.

## Conclusion and future perspectives

Exosomes have recently emerged as promising therapeutic vehicles with several potential advantages over conventional delivery systems in selected applications. These nanoscale carriers exhibit excellent biocompatibility and low toxicity; reports of their ability to cross biological barriers such as the blood-brain barrier (BBB) exist, although this capability is highly dependent on vesicle origin, surface composition and administration route, and remains inconsistent across studies. Exosomes are secreted by various cell types and have shown significant potential as drug delivery systems for immunological, dermatological, cardiovascular and neurological disorders, as well as in vaccine development, tissue regeneration and gene therapy applications. Unlike many synthetic nanocarriers, exosomes provide protection for nucleic acids-including siRNA, mRNA and DNA against intracellular degradative pathways such as endosomal and lysosomal digestion, ensuring successful intracellular delivery.

However, liver, spleen and macrophage-rich tissues represent major sites of exosome sequestration following systemic administration, which significantly reduces the fraction of vesicles reaching intended target tissues. The performance of exosome-based delivery systems can vary substantially depending on vesicle source, purification strategy, administration route and therapeutic cargo, indicating that their advantages over established viral and synthetic vectors may be application-specific rather than universal.

To strengthen the clinical translation of exosome-based therapies, future research should focus on:

- Improving cargo-loading efficiency: Development of novel physical, chemical and bioengineering strategies to enhance the encapsulation of therapeutic molecules, particularly large plasmids and mRNA.
- Enhancing target specificity: Surface modifications, ligand conjugation or receptor-mediated targeting to increase tropism and reduce off-target uptake.
- Scalable production and standardization: Establishment of robust, reproducible and high-yield exosome production and purification protocols suitable for clinical-grade applications.
- Maintaining cargo bioactivity: Optimizing methods to prevent degradation of loaded therapeutics during storage, circulation and cellular uptake.
- Expanding preclinical and clinical studies: Evaluation of exosome therapies in diverse disease models to validate efficacy and safety.
- Combination therapy approaches: Investigating synergistic effects of exosomes with conventional drugs, gene therapy and immunomodulatory strategies for enhanced therapeutic outcomes.

In conclusion, exosomes represent multifunctional, safe and versatile delivery platforms with immense potential in targeted therapy, immunomodulation and gene therapy. Strategic improvements in cargo loading, targeting and production will further accelerate their translation from bench to bedside, paving the way for next-generation precision therapeutics.

## Long descriptions

### Figure 1. Long description

The diagram is split into two main sections.

Left panel: Viral vector.

* At the top, a Viral vector (e.g. lentivirus) binds to a Receptor on the cell membrane.

* An arrow points to the vector being engulfed into the Cytoplasm via an endosome.

* Below this, a label reads Endosome uncoating of vector, showing the vector released from the vesicle.

* An arrow points further down into the Nucleus, where a double-helix D N A strand is shown.

* A vertical arrow labeled Transcription points from the D N A to a single-stranded R N A molecule.

Right panel: Non-viral vector.

* At the top left, a Lipid nanoparticle (S L N or N L C) binds to a Receptor.

* At the top right, an Extracellular vesicle (E V) also binds to the membrane.

* Arrows show both particles entering the Cytoplasm through endosomes.

* Below, the label Endosome uncoating of nanoparticle describes the release of the internal cargo from both the lipid nanoparticle and the E V.

* A horizontal double-headed arrow connects the two released particles, and a central vertical arrow points downward to a final m R N A strand.

Navigate back to Figure 1..

### Table 1. Long description

The table consists of six columns: Product, Regulatory agency, Approval year, Diseases, Mechanism of action, and Limitations/Challenges.

* Luxturna (voretigene neparvovec): F D A, 2017. Diseases: Inherited retinal dystrophies. Mechanism: Delivery of functional R P E 65 gene via A A V 2. Limitations: High cost; applicable only to ocular diseases.

* Zolgensma (onasemnogene abeparvovec): F D A, 2019. Diseases: Spinal muscular atrophy (S M A). Mechanism: Replacement of S M N 1 gene via A A V 9. Limitations: Risk of immune response; extremely high cost.

* Strimvelis: E M A, 2016. Diseases: A D A-S C I D. Mechanism: ex vivo gene transfer to patient’s hematopoietic stem cells using a retroviral vector. Limitations: Complex ex vivo procedure; limited availability.

* Kymriah (tisagenlecleucel): F D A, 2017. Diseases: B-cell acute lymphoblastic leukemia; diffuse large B-cell lymphoma. Mechanism: C A R-T therapy: modification of autologous T-cells targeting C D 19. Limitations: Cytokine release syndrome; complex manufacturing process.

* Yescarta (axicabtagene ciloleucel): F D A, 2017. Diseases: Large B-cell lymphoma. Mechanism: C A R-T therapy: modification of autologous T-cells targeting C D 19. Limitations: Toxicity; high cost.

* Gendicine: C F D A (China), 2003. Diseases: Head and neck squamous cell carcinoma. Mechanism: Delivery of p 53 gene via adenoviral vector. Limitations: Limited efficacy in solid tumors.

* Imlygic (talimogene laherparepvec): F D A, 2015. Diseases: Melanoma. Mechanism: Oncolytic H S V-1 expressing G M-C S F. Limitations: Only for local injection; restricted to accessible lesions.

Navigate back to Table 1..

### Figure 2. Long description

At the center is a large circular cell divided vertically by a dashed line. The left half is shaded pink and contains organelles like the mitochondria and Golgi apparatus. The right half is shaded light blue and features a D N A double helix inside a gear-like structure. Surrounding this central cell are various delivery systems arranged in a circle. Starting from the top and moving clockwise, the labels and structures are as follows.

* Transdermal drug delivery system: A row of blue microneedles.

* Phytosome: A circular lipid bilayer with purple molecules embedded.

* Liposomes: A yellow bilayer sphere with a single lipid molecule highlighted.

* Niosomes: A blue bilayer sphere with a single molecule highlighted.

* Lipid nanoparticle: A yellow sphere with a solid blue core.

* Dendrimer: A highly branched, tree-like molecular structure.

* Hydrogel: A translucent blue triangular mass containing orange fibrous networks.

* A d V: A red icosahedral viral vector with spikes.

* L V forward slash R V: A green circular viral vector with internal genetic material.

* H S V: A pink circular viral vector with a hexagonal core.

* Liquid crystal: A blue cubic lattice of spheres.

* Microsphere: A solid light blue sphere.

* Nanoparticle: A blue sphere with a porous or dotted surface.

* Proniosome: A blue bilayer sphere with a light blue center.

* Ethosome: A blue bilayer sphere with purple oval inserts.

Navigate back to Figure 2..

### Table 2. Long description

The table compares A A V, Lentivirus (L V), Adenovirus (AdV), L N P, Polyplex, and Exosome platforms.

* Payload capacity: A A V is approximately 4.7 kb (ss D N A); L V is approximately 8 kb; AdV is approximately 36 kb; L N P and Polyplex are flexible (si R N A, m R N A, D N A); Exosome is limited (1% to 10% efficiency).

* Transduction/delivery efficiency: High for A A V, AdV, and L N P; Very high for L V; Moderate for Polyplex; Low to moderate for Exosome.

* Immunogenicity risk: Low to moderate for A A V and L N P; Moderate for L V and Polyplex; High for AdV; Low to moderate for Exosome.

* Biodistribution/clearance: A A V is serotype-dependent (liver, C N S); L V is broad and integrates; AdV is broad with rapid clearance; L N P is primarily liver; Polyplex and Exosome accumulate in liver/spleen.

* Repeat dosing: Feasible for L N P, Polyplex, and Exosome; Limited for A A V and L V; Not suitable for AdV.

* Scalability/G M P manufacturing: High for L N P; Moderate for A A V, AdV, and Polyplex; Complex for L V; Low for Exosome.

* Insertional mutagenesis risk: High for L V; Very low for A A V; None for AdV, L N P, Polyplex, and Exosome.

* Clinical approval status: Multiple approved for A A V, L V, L N P, and Polyplex; Limited for AdV; None approved for Exosome (Phase I to II trials ongoing).

Navigate back to Table 2..

### Figure 3. Long description

The diagram is divided into two main sections.

On the top left, a detailed cross-section of a single vesicle shows its internal and surface components. At the center is the Cargo, containing nucleic acids, proteins, and lipids. The surrounding lipid bilayer membrane features several embedded structures: Surface antigen, Adhesion molecules, Tetraspanin, and a Lipid raft.

On the right, a large cell-like structure illustrates the formation pathways.

* The exosome pathway begins at the top with an Early endosome. An arrow points downward to Multivesicular bodies, which contain several small internal vesicles. A subsequent arrow points to the plasma membrane where these small vesicles are released into the extracellular space as Exosomes, labeled with a size range of 30 to 150 n m.

* The microvesicle pathway is shown at the bottom right, where the plasma membrane buds outward directly to release larger vesicles labeled Microvesicles, with a size range of 100 to 1000 n m.

Navigate back to Figure 3..

### Table 3. Long description

The table contains the following data across eight columns:

* Aruna bio: Neural stem cells; AB126 exosome platform; Neurological disorders; Native E V cargo; I V (preclinical models); Preclinical; Proprietary isolation and limited Q C disclosure.

* Ciloa: Recombinant exosomes; Engineered exosome vaccine platform; Viral diseases; Antigen/protein; Injection; Preclinical/early clinical; Engineered loading and scalable recombinant system.

* Evox therapeutics: Engineered exosomes; DeliverEX platform; Rare genetic disorders; R N A/protein therapeutics; I V; Preclinical/clinical pipeline; G M P-oriented scalable E V engineering platform.

* Carmine therapeutics: R B C-derived E Vs; REGENT platform; Gene therapy; R N A/D N A cargo; I V; Preclinical; Enucleated R B C source reduces oncogenic risk.

* Direct biologics: M S C-derived E Vs; ExoFlo; Inflammatory diseases; Native M S C secretome; I V; Clinical use (limited indications); Batch variability noted and M S C-based G M P production.

* Aethlon medical: Filtration-based system; Hemopurifier; Cancer, viral infections; E V depletion/immune modulation; Extracorporeal; Clinical device stage; Device-based C M C framework.

* Kimera labs: M S C-derived E Vs; XoGlo; Wound healing, skin repair; Growth factors plus E Vs; Topical/injection; Commercial/clinical use; M S C-conditioned media and limited standardization.

* Capricor therapeutics: Cardiac M S C E Vs; Exosome platform; Duchenne muscular dystrophy; Protein/R N A cargo; I V; Clinical trials; G M P M S C expansion plus E V isolation pipeline.

* Codiak biosciences: Engineered E Vs; engEx platform; Cancer, C N S diseases; Engineered R N A/protein; I V; Clinical trials; Defined engineering plus Q C biomarker panels.

* Exopharm Pty Ltd: Purified E Vs; LEAP technology; Multiple indications; Purified E V fractions; I V/topical; Preclinical/early clinical; Scalable purification plus E V enrichment Q C.

* ReNeuron: Stem cell E Vs; ExoPrO platform; Neurodegenerative diseases; Native E V cargo; I V; Clinical pipeline; Stem cell-derived G M P manufacturing.

Navigate back to Table 3..
